# In Vitro Scanning Electron Microscopic Study on the Effect of Doxycycline and Vancomycin on Enterococcal Induced Biofilm

**Published:** 2010-05-20

**Authors:** Krishnaraj Somayaji, Shashi Rashmi Acharya, Indira Bairy, Peralam Yegneswaran Prakash, Muddanna Sakkattu Rao, Nidambur Vasudev Ballal

**Affiliations:** 1. Department of Conservative Dentistry and Endodontics, Manipal College of Dental Sciences, Manipal University, Manipal, Karnataka, India.; 2. Department of Microbiology, Kasturba Medical College, Manipal, Karnataka, India.; 3. Department of Anatomy, Faculty of Medicine, Kuwait University, Safat, Kuwait.

**Keywords:** Biofilm, Doxycycline, Enterococcus faecalis, Root canal, Vancomycin

## Abstract

**INTRODUCTION:**

Enterococcus (E) faecalis bacteria adhere to dentine of teeth root canals to form the biofilm. E. faecalis has been shown to be resistant to antibiotics. This in vitro study aimed to investigate the efficacy of vancomycin and doxycycline in inhibiting E. faecalis biofilm formation.

**MATERIALS AND METHODS:**

A total of 60 extracted human teeth were incubated with E. faecalis (ATCC 35550 strain) for 45 days to allow biofilm formation. The teeth were equally divided into six groups (n=10): 1) positive control, 2) negative control, 3) doxycycline alone 4) doxycycline with filing, 5) vancomycin alone, 6) vancomycin with filing. The relevant canals were irrigated with 4µg/mL of either vancomycin or doxycycline antibiotic. Teeth were processed for scanning electron microscopy (SEM). Areas of biofilm remaining in the canals after antibiotic treatment were measured with Scion image analysis software using the SEM images.

**RESULTS:**

Vancomycin is more effective in reducing the overall biofilm area compared with doxycycline; moreover filing after antibiotic administration increased this effect.

**CONCLUSION:**

We can conclude that vancomycin had greater efficacy than doxycycline for inhibiting and reducing E. faecalis biofilms growth in root canals. However, it failed to completely eliminate biofilm formation.

## INTRODUCTION

The most common reasons for endodontic failures are related to inadequate or problematic instrumentation and bacterial resistance to endodontic therapy [1]. Viable microorganisms that remain after root canal disinfection and treatment significantly contribute to endodontic failures [2]. In one study a staggering 88% of root canals treated contained residual infection [3]. In these teeth, the presence of Enterococcus (E) faecalis has been repeatedly reported [4][5]. Moreover, if this bacterium is isolated during the time of root filling or after endodontic treatment, frequencies of treatment failures are higher [5]. E. faecalis is the dominant post-treatment microbe in apical periodontitis and has often been isolated from the root canal in pure cultures. Its pathogenicity in endodontic infections is well documented. It has been reported that the prevalence of this bacterial infection ranges from 24% to 77% in asymptomatic persistent endodontic infections [6].

The tooth root canal is an extraordinary micro-environment for the attachment of several microbial species to the dentin, and the basis to form dense biofilms [3]. Though E. faecalis is easily destroyed when grown in-vitro; it displays resistance in the root canal system possibly by activating virulence factors within the biofilm. Bacteria that are part of a biofilm are 2 to 1000 times more resistant than the corresponding bacteria in planktonic form [7]. Biofilms are complex bacterial communities embedded in a polysaccharide matrix, which makes them more resistant to phagocytosis, antibodies and antibiotics [8]. Biofilm bacteria may show phenotypical changes which enhances their resistance to antimicrobial drugs. Matrix layer may prevent direct contact of most of the antimicrobial agents with microorganisms. Subpopulation of micro-organisms may form a phenotypic state called persister that is highly protected [9]. The majority of studies discussing the efficacy of antibiotics on biofilm bacteria provide contradictory antimicrobial strategies, possibly due to difference in methodology applied. In a study on 21 different strains of E. faecalis isolated, all the strains were susceptible to vancomycin; however, only 85.7% were susceptible for doxycycline [10].

Therefore, as the environmental growth conditions may vary between biofilms and strains of planktonic microbes, this may be why there is a difference in resistance to antimicrobial drugs. Though sodium hypochlorite is very effective irrigant against E. faecalis biofilms, in certain cases where isolation is not possible the use of sodium hypochlorite may lead to risk of tissue injury. Hence, this study was aimed to investigate the efficacy of vancomycin and doxycycline on removal of biofilm formed with in the extracted human teeth root canals by E. faecalis within human root canals.

## MATERIALS AND METHODS

### Preparation and processing of teeth

Single-rooted extracted human maxillary incisor teeth (n=60) were collected from dental clinics in College of Dental Sciences, Manipal University, Manipal and adjoining local dental clinics for this study. The protocol was approved by the institutional ethical committee of Manipal University. The teeth were thoroughly cleaned and treated with hydrogen peroxide 10% to remove the attached debris followed by washing with distilled water. Teeth were allowed to dry at room temperature.

After preparing the access cavity, working length was determined by a periapical radiograph 1 mm short of the radiographic apex. Coronal third of the canal was flared with Gates Glidden drills in the sequence of No. 1, 2 and 3. The canals were prepared up to size #25 K-file (Dentsply, PA, USA), 1 mm short of apical foramen. Step-back preparation was completed up to K-file size #50. During instrumentation of the root canals, canals were irrigated with sodium hypochlorite 5.25% alternating with EDTA (RC-prep, Premier Dental Products, USA). This was performed to simulate clinical retreatment conditions. Teeth were then decoronated at cementoenamel junction to achieve a uniform root length (~17 mm). The canals were then dried at room temperature. Two longitudinal grooves were made along the entire length on opposite sides of the outer surface of the teeth to act as a guide for the subsequent splitting of the tooth into two halves [11]. The teeth were the finally sterilized using gaseous ethylene oxide.

### Experimental groups and Antibiotic treatment:

All the 60 sterilized teeth were assigned into six experimental groups (n=10).

***Group I: Negative control:*** teeth in this group did not receive any bacterial treatment.

Remaining 50 sterilized teeth were incubated in live suspensions of biofilm forming strain of E. faecalis (ATCC 35550 strain) in cryovials for 45 days at 37˚C with brain heart infusion broth. On the 46th day these teeth were randomly divided into the 5 groups (n=10) (Group II to VI).

***Group II: Positive control:*** No irrigation was used and teeth were processed for fixation for scanning electron microscopic examination.

***Group III:Doxycycline:*** root canals were irrigated with 4µg/mL of doxycycline (Hi Media Laboratories Pvt. Ltd., Bombay, India) [12]. Canals were irrigated thrice with a 5mL sterile syringe at 10-min intervals, so as to keep irrigation sequence consistent between instrumented and non-instrumented groups.

The specimens were then kept in phosphate buffer saline (PBS) at 37˚C for twelve hours and then processed for scanning electron microscopy (SEM).

***Group IV:Doxycycline with filing:*** the root canals of teeth were irrigated as in the group III, and then filed with #30 and #35 size K-file.

***Group V:Vancomycin:*** root canals in this group were irrigated three times (10 min intervals) with 4µg/mL of vancomycin (Hi Media Laboratories Pvt. Ltd., Mumbai, India) using a 5 mL sterile syringe, as the previous groups [12]. The specimens were then kept in PBS at 37˚C for twelve hours and then processed for SEM.

***Group VI:Vancomycin with filing:*** teeth in this group were irrigated with vancomycin as in group IV, and filed with #30 and #35 size K-file and then processed for SEM.

### Scanning electron microscopy:

Teeth in all the above groups were processed for SEM. The SEM used for the study was manufactured by JEOL Ltd. 1-2, Musashino, 3-chome Akishima Tokyo 196-8558, Japan. The SEM was performed to visualize the remaining biofilm after antibiotic treatment alone or antibiotic treatment followed by filing. With the help of a hammer and a sharp chisel, teeth were split into two halves along the longitudinal grooves cut previously [11]. Each of the half roots was gently washed in 0.5 M potassium phosphate buffer, at pH 7.2, and 5˚C. The roots were then fixed in 2% glutaraldehyde at 5˚C for 20 hrs, washed with phosphate buffered saline (PBS) for 15 min and post fixed for 12 hours at 5˚C in 1% (wt/vol) osmium tetroxide solution. PBS was used as the final wash. A series of ascending grades of acetone (30%, 60%, 80% and 100%) were utilized for 10 min each to dehydrate the specimens. The roots were then dried by keeping them in slanting position in the oven at 37˚C for 5 hours. Each root was mounted and coated with a 200 a layer of gold palladium. Root canals were observed using a JEOL JSM-35 CF SEM. Photographs were taken from randomly selected fields and stored in a compact disc for further analysis using the Scion image analysis software.

### Scion image analysis:

The SEM images taken at 20KV ×2000 magnification were opened in the scion image analysis software (NIH image software, Scion Corporation, USA). From each teeth 10 randomly selected images were considered for image analysis (n=6 teeth in each group). The software was calibrated using the scale bar on each image. The total area of the image and the area occupied by the biofilm were measured using the area option in the software. In this way the data percentage of the total area occupied by the biofilm was calculated.

### Data analysis

Data were expressed as mean ± standard errors of means and analyzed by one-way ANOVA, followed by Bonferroni’s test using GraphPad InStat software version 14.0.

## RESULTS

During the bacterial incubation period, E. faecalis (ATCC 35550 strain) formed well developed biofilms inside the root canals.

**Figure 1:**
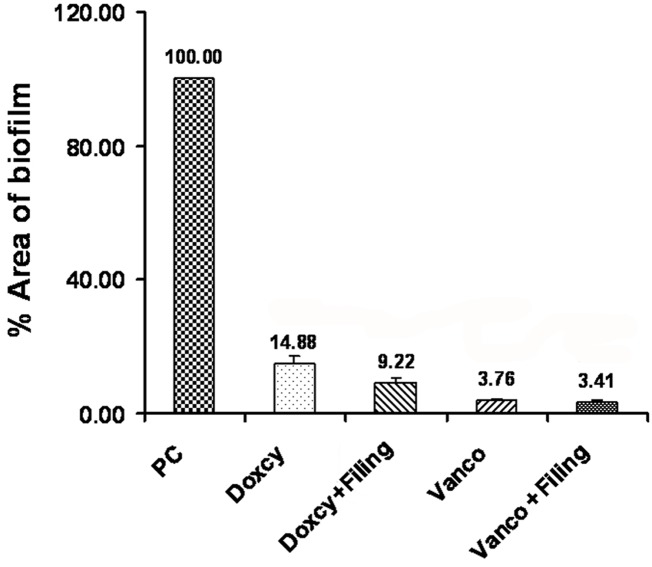
Area of biofilm formed in positive control (PC), and biofilm remained after irrigation with doxycycline (Doxcy), doxycycline followed by filing (Doxcy + filing), vancomycin (Vanco), vancomycin followed by filing (Vanco + filing) groups.

**Figure 2:**
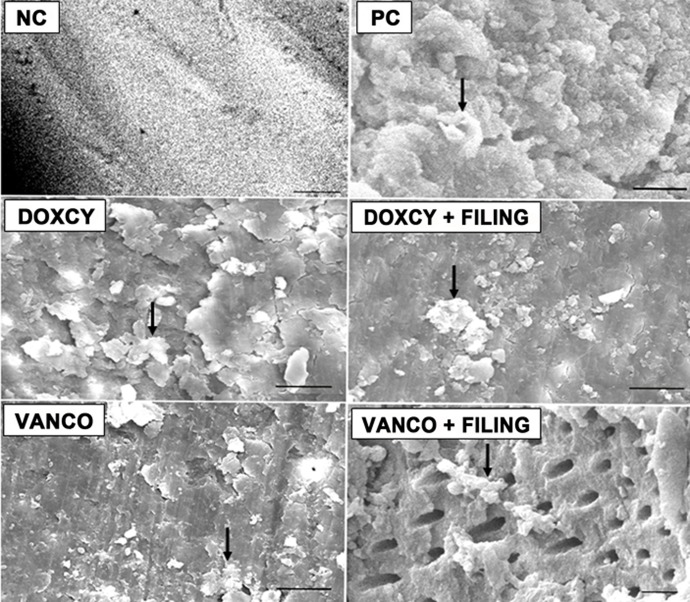
SEM image of biofilm (Arrows) in positive control (PC), doxycycline (Doxcy), doxcyline and filing (Doxcy + filing), vancomycin (Vanco), vancomycin and filing (Vanco + filing) groups. Negative control photomicrographs are taken under lower magnification compared to the other groups.

The biofilm areas that remained after various treatments as measured by the scanning electron micrograph are shown in Figure 1 and Figure 2.

Total biofilm area formed in the positive control group was valued at 100%. The results of our study revealed that treatment with doxycycline reduced the percentage of area of biofilm formed to 15% (14.88 ± 2.51%) compared with the positive control group (P<0.001).

Treatment with doxycycline and filing further reduced the formed biofilm area to 9.00% (9.22 ± 1.58%) (P<0.001).

Similarly, data showed a significant reduction (P<0.001) in the percentage biofilm area with vancomycin (Group 5) to 4% (3.76 ± 0.57 %) (P<0.001). Treatment with vancomycin and filing further reduced the biofilm area to 3.00% (3.41 ± 0.39 %) of the positive control (P<0.001).

Irrigation with doxycycline alone and doxycycline with filing did not demonstrate significant difference. Similarly, irrigation with vancomycin alone and vancomycin with filing was not significantly different. However, vancomycin significantly removed biofilm when compared with the doxycycline group (P<0.001).

## DISCUSSION

The E. faecalis is the microbial flora found in resistant apical periodontitis and can threat other sites with infection [13]. It is often isolated in apical periodontitis retreatments, but is seldom found in primary endodontic infections [14]. It is one of the few bacteria isolated as a monoculture from root canal and is more resistant to root canal medicaments and antibiotics. Their exceptional resistance and survival against antimicrobial agents and root canal therapy is due to their phenotype modification within the biofilm [15]. Several studies on biofilm models analyzed intracanal medicaments; however, those which can propose antimicrobial strategies are limited [16]. Some studies reported the variable thickness of biofilms on the disc and root canal surfaces when incubated in the bacterial suspension [1][17][18][19]. Therefore, E. faecalis biofilm formation and growth is dependent on the type of bacterial isolate, environmental and nutritional conditions. In our study, we observed well developed E. faecalis (ATCC 35550) biofilm in the human root canals by 45 days of incubation in live bacterial suspension. Microbial ability to form biofilms and their resistance to drugs are species dependant [20].

Collagen binding protein (serine), ACE and possibly gelatinase are potential virulence factors that enable E. faecalis to bind to dentine [21]. Heterogeneous colonies possess a distinct advantage over homogenous ones in production of surface biofilms [22].

Biofilm formation takes place in stages and E. faecalis with its capacity to induce apatite re-precipitation in mature biofilms may persist after root canal treatment. Biofilm is an effective microbial defense mechanism against host defenses and antimicrobial agents [23].

The micro-organisms that remain in irregular spaces of root canal system are the main etiological factor for the failure of root canal disinfection and treatment [24]. The current techniques of root canal debridement leave many areas of the root canal system untouched by instruments [25]. Thus root canal irrigation is needed to aid in debridement of the canal.

E. faecalis has biofilm forming capacity, dentine collagen binding property, substantial virulence factors and ability to survive in critical environment, making it resistant to endodontic therapy [21][26][27].

MTAD BioPure and Tetraclean have been proposed as final rinse in endodontic treatment. These irrigants are mixtures of doxycycline, citric acid, and a surfactant. This study irrigated with vancomycin and doxycycline solutions instead of sodium hypochlorite; a tissue irritant. Doxycycline, readily attaches to dentine and is subsequently released without losing its antibacterial activity [10][25]. This property creates a reservoir of active antibacterial agent, which is released in a slow and sustained manner from the dentin surfaces. The most effective concentration of doxycycline hydrochloride that removed intracanal smear layer was 100mg/mL [28]. Doxycycline possesses antimicrobial substantivity, but does not dissolve tissue and therefore should be considered as the final rinse, prior to root canal obturation [25]. Most previous studies used antibiotics/medicaments for longer period than clinically practical [1]. In the present study the biofilm was developed after the initial enlargement of the canal to simulate retreatment conditions. Filing was performed to assist the efficient eradication of biofilms. We found vancomycin to be statistically more effective (P<0.001) in reducing E. faecalis biofilm; this concurs with a previous study [10]. Vancomycin irrigation with filing showed better results than vancomycin irrigation alone; however, the difference was not statistically significant. Doxycycline with filing showed better results in the eradication of biofilm than the doxycycline alone, but the difference was not statistically significant. This may be due to inaccessibility of the file to reach all parts of the canal during the filing process in both cases. Our study showed that vancomycin is significantly superior to doxycycline in reducing E. faecalis biofilm. Both antibiotics appear more effective in eradicating the biofilm than filing. Low concentrations of sodium hypochlorite mainly dissolve necrotic tissue; at higher concentration it dissolves both necrotic and vital tissue, a generally undesirable effect.

## CONCLUSION

Both vancomycin and doxycycline were effective in reducing biofilms developed in vitro in the human root canal system; however, vancomycin was statistically more effective. Irrigation with antibiotics combined with filing showed slightly better results than without filing; however this was not statistically significant. Though our procedure failed to eradicate the biofilms completely, prolonged antibiotic irrigation and filing in a future study may achieve this goal.
